# Immobilization Techniques in the Fabrication of Nanomaterial-Based Electrochemical Biosensors: A Review

**DOI:** 10.3390/s130404811

**Published:** 2013-04-11

**Authors:** William Putzbach, Niina J. Ronkainen

**Affiliations:** 1 Department of Cell & Molecular Biology, Northwestern University, 303 E. Chicago Avenue, Chicago, IL 60611, USA; E-Mail: williamputzbach2012@u.northwestern.edu; 2 Department of Chemistry and Biochemistry, Benedictine University, 5700 College Road, Lisle, IL 60532, USA

**Keywords:** biosensors, carbon nanotubes, electrochemical detection, enzyme-coupled electrochemical biosensors, enzyme immobilization, gold nanoparticles, graphene

## Abstract

The evolution of 1st to 3rd generation electrochemical biosensors reflects a simplification and enhancement of the transduction pathway. However, in recent years, modification of the transducer with nanomaterials has become increasingly studied and imparts many advantages. The sensitivity and overall performance of enzymatic biosensors has improved tremendously as a result of incorporating nanomaterials in their fabrication. Given the unique and favorable qualities of gold nanoparticles, graphene and carbon nanotubes as applied to electrochemical biosensors, a consolidated survey of the different methods of nanomaterial immobilization on transducer surfaces and enzyme immobilization on these species is beneficial and timely. This review encompasses modification of enzymatic biosensors with gold nanoparticles, carbon nanotubes, and graphene.

## 1. Introduction

**Figure 1.**
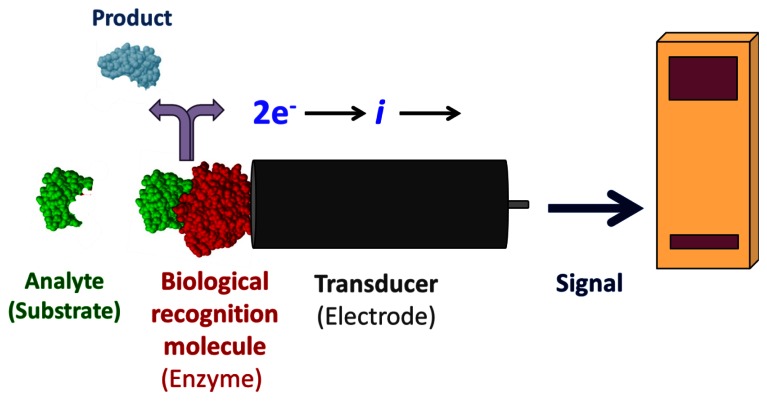
A typical design of an enzyme modified electrochemical biosensor.

Enzyme-coupled electrochemical biosensors are based on the detection of an electric signal produced by an electro-active species, either produced or depleted by an enzymatic reaction [1]. The relatively simple layout consists of a bio-recognition layer of enzymes attached to a working electrode, a transducer (Figure 1). Enzymes are optimal biorecognition molecules because they provide excellent selectivity for their targeted substrate and have high catalytic activity. At the same time, enzymes are the shortest lived component of these biosensors because they gradually lose activity, thereby determining the lifespan of the biosensor. While the enzyme layer catalyzes the production or depletion of an electro-active species, a voltage is applied to the electrode in amperometric sensors, which induces redox reaction of the electro-active species—generating a signal [1]. This electrical signal correlates to the concentration of analyte in the sample. A change in electrode potential can also be used as the measurable transducer response in potentiometric sensors. Finally, a signal processor connected to a transducer collects, amplifies, and displays the signal. Using electrodes as signal transducers in biosensors is quite popular because of the high sensitivity and operational simplicity of the method [1]. Electrochemical detection also offers additional selectivity as different electroactive molecules can be oxidized/reduced at different potentials. Electrochemical detection is also compatible with most modern miniaturization/microfabrication methods, has minimal power requirements, and is independent of sample turbidity and color. Most enzyme-based electrochemical biosensors do not require extensive instrumentation making them relatively inexpensive. Enzyme electrodes are used in many point-of-care and clinical applications for a broad range of analytes.

Electrochemical biosensors are also popular due to their low-cost and relatively fast response times. An ideal biosensor has a high S/N ratio and a low detection limit [1]. Detection limit is often defined as three times the standard deviation of the blank. Having a broad linear range for detection of the analyte is also desirable. There are, however, disadvantages with electrochemical sensors, particularly when coupled to an enzymatic reaction. The main challenge in developing these electrochemical biosensors has been overcoming the often inefficient electron transfer between the enzyme and the electrode surface [2]. This is generally due to the redox active site being buried deep within the enzyme and the inability of the enzyme to orient itself favorably with respect to the electrode surface for fast and efficient electron transfer [2]. Other challenges associated with electrochemical biosensors that are being addressed by ongoing research include non-specific binding and sometimes limited ability to function adequately in real-world samples due to electrode fouling or poor selectivity for the analyte in a complex sample matrix. There are also continuous efforts to miniaturize the biosensors and make them biocompatible for *in vivo* measurements. Biocompatibility is often important since blood and other biological fluids are the most common sample matrices for enzyme electrodes in clinical chemistry applications. Many blood components may rapidly foul the electrode unless special consideration is given to optimizing the sensor's outermost surface properties and selective permeability of analytes [1]. The main applications for electrochemical biosensors are in food and beverage quality control, security, environmental monitoring, bioprocessing, and most commonly in health care. Determination of glucose in blood continues to be the most dominant and most studied application of electrochemical biosensors and as such is the most successful commercial application of enzyme-coupled biosensors [1].

This review focuses on the use of various nanomaterials in electrochemical biosensors, specifically, how enzymes are immobilized on such modified electrodes and how the nanomaterials and incorporated into the sensor devices. Nanomaterials are defined as materials with dimensions smaller than 100 nm and include metallic nanoparticles made of gold and silver as well as carbon nanomaterials. Combining the bioselectivity and specificity of enzymes with the numerous and advantageous chemical and physical properties of nanoparticles has allowed the development of a whole new subset of sensitive biosensor devices. In addition, the modification of enzymatic biosensors with gold nanoparticles, carbon nanotubes, and graphene will be discussed. First, a brief history of the evolution from first to third generation electrochemical biosensors is outlined, with glucose being used as an example of an analyte.

## 2. Evolution from 1st to 3rd Generation Biosensors

The first glucose biosensor was developed by Clark and Lyons of the Cincinnati Children's Hospital in 1962. Their sensor used glucose oxidase (GOx) entrapped over an oxygen electrode by a semipermeable membrane to select for β-D-glucose in the presence of oxygen gas [3]. The oxygen consumption as it reacted with protons and electrons to produce water was detected by the electrode as a change in potential. The first commercially available glucose sensor was sold by the Yellow Springs Instrument (YSI, Yellow Springs, OH, USA) for analysis of whole blood samples. Although many improvements have been made in glucose and other biosensors, the same general structure for constructing enzyme electrodes is still used today.

**Figure 2.**
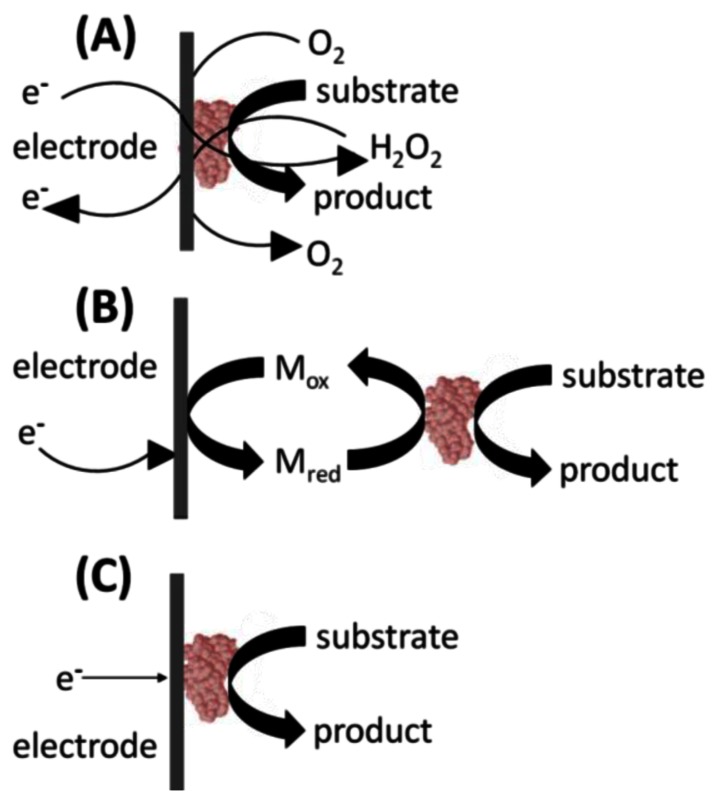
The evolution from 1st to 3rd generation electrochemical biosensors. The figure highlights modifications in the biosensor layout with each generation using glucose sensors as an example.

In the 1st generation glucose biosensor, the trapped GOx would oxidize β-D-glucose to β-D-gluconolactone, with a simultaneous reduction of FAD to FADH_2_ (Figure 2(A)) [1]. Next, the FAD would be regenerated from FADH_2_, using dissolved O_2_ to produce H_2_O_2_. Finally, an applied voltage would induce oxidation of the H_2_O_2_ at the electrode surface, producing an electric signal. Unfortunately, the 1st generation biosensor layout harbors several shortcomings. First, the active site and the FAD prosthetic group are buried deep within the enzyme, severely restricting the diffusion of reagents. Moreover, the Marcus theory states that electron transfer decays exponentially with increasing distance [4]. The active sites of enzymes are typically buried within the protein shell [5]. Therefore, the ability of electrons to “escape” the confines of the enzyme to the electrode surface is restricted. Second, O_2_ has a limited solubility in aqueous media. It is, therefore, the limiting reagent, leading to a detrimental O_2_ deficiency at higher glucose concentrations and changes in sensor response. This ultimately results in narrow linear range for the glucose measurements [6]. Additionally, the partial pressure of O_2_ is difficult to control, leading to fluctuating amounts of the reagent present in the biosensor's immediate environment [6]. Finally, a high voltage must be applied to induce oxidation of hydrogen peroxide at the electrode surface. This will lead to redox of interfering electro-active species commonly present in the blood sample matrix, such as ascorbic acid, paracetamol, and uric acid [6]. In turn, this leads to a background signal from the other electroactive species which erodes the S/N ratio and the detection limits. Fortunately, interference due to electroactive species has since been minimized by including selectively permeable membranes such as cellulose acetate or Nafion between the sample and the enzyme coated electrode. The applied detection potentials have also been reduced to 0–0.2 V (*vs.* Ag/AgCl) to avoid the reduction-oxidation reactions of the interfering species [6].

2nd generation biosensors addressed many of the 1st generation biosensor issues with the incorporation of a synthetic mediator—an electron shuttle molecule—to replace dissolved O_2_ in the production of H_2_O_2_ [6]. Direct electron transfer is not possible without including some sort of mediators to facilitate the transfer because the FAD redox center of GOx is buried inside a thick protein layer resulting in kinetically slow electron transfers [7]. In the 2nd generation biosensor layout, the mediator_Ox_ regenerates the FAD, with a simultaneous self-reduction (Figure 2(B)). Then the mediator_Red_ is regenerated at the electrode surface, producing an electric signal. Ideal mediators react rapidly with the reduced enzyme, have low solubility in aqueous sample environment, are chemically stable in reduced and oxidized forms, are nontoxic, and have good electrochemical properties (*i.e.*, low detection potential) [7]. The mediator may be dissolved in the electrolyte solution to facilitate its mass transport between the electrode surface and the enzyme active site. Mediators such as poly(vinylimidazole) and poly(vinylpyridine) linked with osmium-complex electron relays provided close proximity for the redox center of the polymers and the FAD redox center of the enzymes resulting in fast sensor response and high current output [7]. Their use in 2nd generation biosensors also eliminated the problems associated with oxygen, including O_2_ deficiency and a fluctuating partial pressure. Synthetic mediators are much more accessible in aqueous media than the O_2_, and therefore, address limited diffusion rates associated with 1^st^ generation sensors. Finally, mediators are readily regenerated at lower applied voltages, eliminating the background signal from interfering species. However, some 2nd generation biosensors suffered from leaching of the synthetic mediators from the biosensor over time. For this reason, the use of soluble mediators is unfeasible in biosensors designed for *in vivo* use.

3rd generation biosensors involve “wiring” an enzyme to the electrode by co-immobilizing the enzyme and mediator directly onto the electrode surface or into an adjacent matrix such as a conductive polymer film [6]. The immobilized mediators act as non-diffusion redox relay stations, effectively facilitating the transport of electrons from the enzyme active site to the electrode (Figure 2(C)). In some cases, direct electrical contact can be established between the enzyme and the electrode thus greatly increasing the efficiency of the electron transfer. For these 3rd generation biosensors, immobilized mediators allow efficient electron transfer, resulting in a higher current density. Close proximity of the enzyme and the mediator to the transducer surface minimizes the electron transfer distance thereby resulting in faster response times. Because they are immobilized, mediators cannot escape the biosensor film and leach into the surroundings thereby allowing sensor use for *in vivo* measurements. The applied electrode can be operated at the desired voltage, eliminating background interference. This design also lends itself to repeated and prolonged measurements because there are no reagents to replace. Ferrocene derivatives have been co-immobilized with glucose oxidase into various types of matrices [8].Enzyme and mediator immobilization methods include the layer-by-layer deposition of polyelectrolytes, creating hydrogels and electropolymerization in the presence of the enzyme and the mediator to trap them at the electrode surface [8].

The evolution from 1st to 3rd generation reflects an effort to produce an efficient and selective transduction pathway—one that provides a rapid, amplified analyte signal and minimal background interference. Several successful schemes have been described for establishing a close contact between the enzyme and the electrochemical transducer without interfering with substrate access to the enzyme's active site or adversely altering the overall conformation of the enzyme which in turn may affect its biocatalytic activity. However, these advancements do relatively little to improve the intrinsic properties of the transducer itself. By modifying the transducer, it is possible to further enhance the biosensor's selectivity, increase the S/N ratio, and lower the detection limit. In recent years, electrode modification with nanomaterials such as gold nanoparticles or carbon nanotubes has shown a lot of promise. Recent studies have shown their ability to provide a friendly platform for immobilizing enzymes and further improve electron transfer between the redox center of the enzyme and electrode thereby resulting in faster response times and often higher sensitivity [9]. Often, the immobilization of enzymes also improves their stability by minimizing enzyme unfolding. Therefore, it is beneficial to investigate the methods by which these nanomaterials are used in biosensors, specifically, how enzymes are immobilized on such modified electrodes. The most common physical and chemical enzyme immobilization schemes onto biosensors utilizing nanomaterials such as gold nanoparticles, carbon nanotubes, and graphene will be described.

## 3. Gold Nanoparticles (GNPs)

### 3.1. Characteristics

Colloidal gold is one of the most studied nanomaterials available for biosensors [10]. It is manufactured from small octahedral units called primary units. The size and morphology of the nanoparticles can be manipulated, depending on the synthesis method employed [11]. The gold nanoparticles are usually stored in an aqueous solution. Metal nanoparticles are attractive for construction of biosensor devices due to their ability to enhance the amount of immobilized biomolecules incorporated in a sensor.

GNPs have many advantageous qualities for a variety of biosensing applications. Li *et al.*, describe three such applications: electrochemical biosensors, optical biosensors, and piezoelectric biosensors [11]. As described above, nanomaterials can be used to modify the surface of the electrode. Extensive studies have shown colloidal gold to be promising candidate for modifying electrochemical biosensors. The general layout is to attach or deposit the GNPs onto the electrode. This modified electrode can then host the bio-recognition layer. GNPs introduce many advantages to these sensors, encompassing their ability to provide a friendly and efficient loading platform for immobilizing enzymes and further improve electron transfer between the active site and electrode. Frequently, adsorption onto bulk surfaces results in protein denaturation and decreased performance [12]. Because of colloidal gold's high biocompatibility and surface free energy, enzymes retain their bioactivity, and enzyme loading increases [13,14]. For biosensors, diffusion rates can adversely affect the magnitude of signal. If an enzyme's active site is deeply buried, then the movement of electrons, between the active site and electrode, and reagents will decrease, resulting in a smaller signal. Modification with colloidal gold nanoparticles affords the attached enzyme more freedom of orientation, weakening this insulating protein layer which covers the active site, decreasing the effects of the Marcus theory and increasing diffusion of necessary species [15,16]. Willner and co-workers studied the electron transfer turnover rate of a reconstituted bioelectrocatalyst using GNPs [17]. They conducted a comparative study between GOx-based glucose sensors—one using O_2_ as the electron shuttle molecule (similar to 1st generation) and one using GNPs. The results indicated that GNPs imparted a much higher electron transfer rate than the more primitive layout. The former achieved an electron transfer rate of 700 electrons per second, while the GNP-modified biosensor achieved approximately 5,000 electrons per second. Moreover, colloidal gold can be easily manipulated into a variety of morphologies and sizes, allowing scientists to optimize the enzymes' microenvironment on the electrode surface. Because of gold's excellent conductivity properties, direct electron contact between the active site and electrode can be established. The first account of this direct electron transfer, using GNPs, was reported by Natan *et al.*, in 1996 [18]. Although gold is an inert metal, recent studies have found that the high surface-volume ratio and surface properties [19,20] and quantum-scale dimensions [21] provide colloidal gold with enhanced catalytic activity. This virtually eliminates the need for overpotentials. Finally, because of their small size, nanoparticles have a high surface-volume ratio, leading to more efficient enzyme loading [22,23].

Given these advantages, a collective survey of enzyme immobilization onto these GNPs will be very beneficial. There are four basic methods of enzyme immobilization: physical adsorption, chemical adsorption, self-assembling monolayers (SAMs), and co-modification with electrode component matrix.

### 3.2. Immobilization of Enzyme onto GNPs

#### 3.2.1. Physical Adsorption

**Figure 3.**
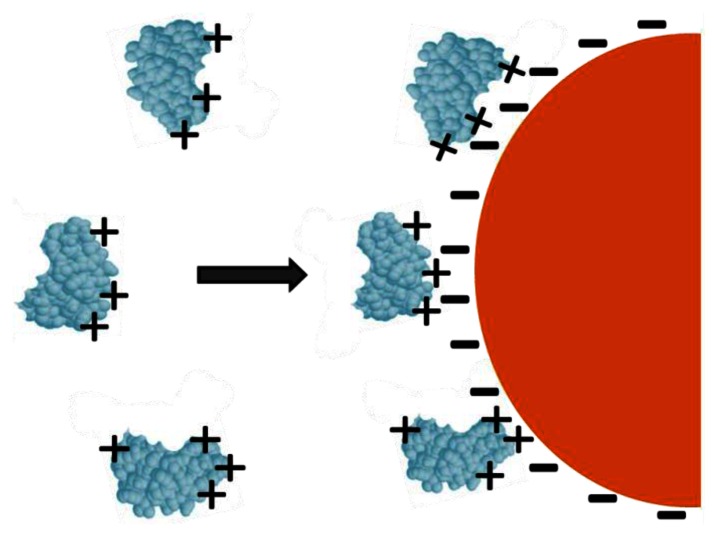
Electostatic adsorption of enzymes directly onto gold nanoparticles. This immobilization method is simple and fast but some enzymes attach to the nanomaterial in unfavorable orientations that decrease their activity.

Physical adsorption is a simple and quick method for manufacturing enzymatic biosensors. It involves reducing the gold nanoparticles with a negatively charged ligand such as citrate. The reduced gold nanoparticles are then allowed to associate with the ligand, insulating the GNPs from electrostatic repulsion and offering it stability. The resulting citrate layer imparts a negative charge onto the colloidal particle surface. Positively charged amino acid residues allow enzymes in solution to be electrostatically adsorbed on the surface by merely dipping the modified electrode into the solution (Figure 3). Although this method has the benefit of speed and simplicity, unfavorable orientations and decreased functionality are likely [12].

#### 3.2.2. Chemical Adsorption

**Figure 4.**
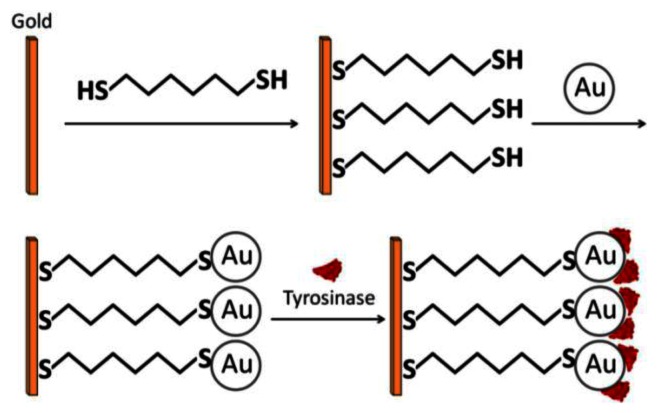
Immobilization of tyrosinase on a gold nanomaterial containing biosensor via chemisorption and covalent attachment.

Chemical adsorption involves direct covalent binding between the enzyme and the electrode surface—the colloidal gold surface. Chemisorption is achieved via covalent interaction between the –SH groups of the cysteine residues and Au on the GNP surface [24,25]. Liu *et al.*, combined the advantages of self-assembly technique (SAMs which are discussed next) and the strong adsorption properties of –SH and Au to construct an economical, simple, and fast enzymatic biosensor for phenolic compound detection, utilizing tyrosinase (Figure 4) [26]. A clean gold electrode (GE) was prepared and incubated in an ethanolic solution containing 1,6-hexanedithiol (HDT) for two hours. Next, it was incubated in a colloidal gold solution, producing a simple monolayer of GE/HDT/GNP. Finally, the modified electrode was dipped in a protein solution of tyrosinase and incubated for 12 hours. Cyclic voltammetry revealed a narrow sigmoidal curve and a wait period of ten seconds to reach 95% of steady-state current. From the voltammetric results, the authors concluded that the catalytic current was mainly due to direct electron transfer from the active site to the electrode.

However, as with all adsorption methods, chemisorption is a non-specific immobilization procedure. Indeed, unregulated covalent binding of an enzyme to a surface can potentially restrict the active site or denature the enzyme. It was therefore, necessary to develop site-specific covalent immobilization methods [12].

**Figure 5.**
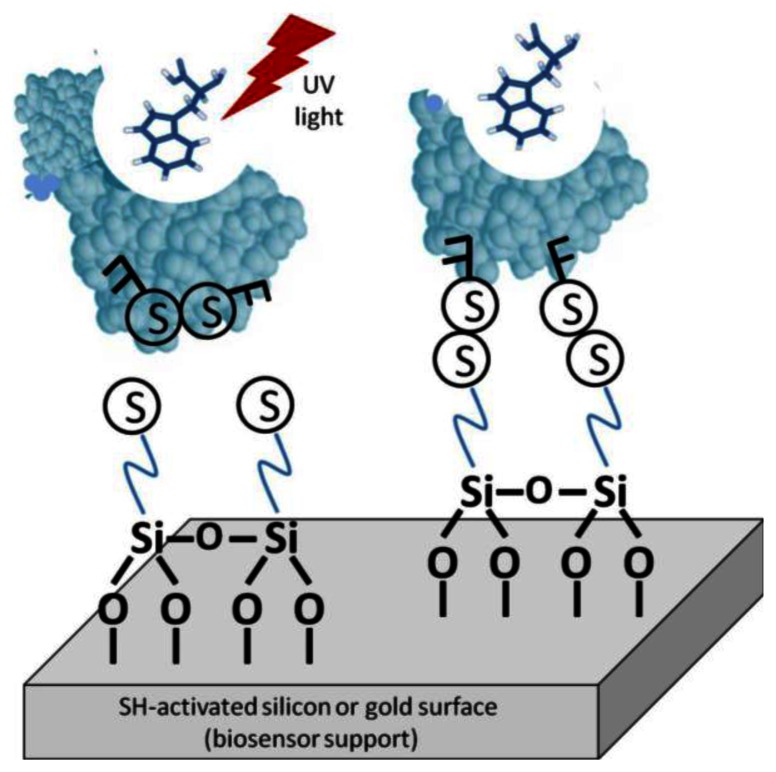
Light-assisted immobilization of proteins onto biosensor support using thiol attachment chemistry.

One such method is light-assisted immobilization, a recently developed technique that allows greater control of the enzymes' orientation via thiol groups (Figure 5). It relies on selective reduction of disulphide bridges, within the enzyme, that are adjacent to aromatic amino acid residues by irradiation with UV light in the 270–300 nm range. The freed –SH groups can then undergo covalent binding with the gold surface [27]. Snabe *et al.*, immobilized a major histocompatibility complex (MHC class I) to a sensor surface using light-assisted immobilization [28]. The authors verified the functionality and accessibility of the peptide/T cell-binding site in the immobilized species. With this method, an intimate understanding of the protein's structure is necessary. The disulfide bridges must be in the correct location on the biomolecule to be utilized. Furthermore, it must be verified that disulfide degradation will not compromise the enzyme's stability or bioactivity.

For comparison, immobilization was also done without the laser TIRF system [28]. The correct orientation was analyzed by the binding of monoclonal anti-HLA class 1 antigen-Fluorescein conjugate to the MHC complex. Binding was measured using fluorescence emission spectroscopy at 525 nm (excitation at 495 nm). The background immobilization was about 46% on average relative to bound mAb conjugate on MCH molecule.

Another site-directed covalent method is manufacturing a modified enzyme with a genetic tag. The processes by which an enzyme is genetically modified or tagged are complex and will not be discussed in detail here. In short, the modified enzyme contains artificially added residues or complexes that can be allocated to a specific area on the enzyme. By covalently binding to the modified region, specific orientation can be achieved. A recent study modifies an enzyme with a metal-binding site, allowing for reorientation. Madoz-Gurpide *et al.*, immobilized ferredoxin:NADP^+^ reductase onto a modified gold electrode by introducing a genetically engineered metal binding site on a specific region of the protein surface [29]. The gold electrode was covered with a self-assembled monolayer of thiols appended with nitrilotriacetic acid groups complexed with metal transition ions. Two mutants were designed to have a histidine pair (His-X_3_-His) on surface-exposed α-helices located in one of the two protein domains. The two mutant enzymes demonstrated differences in enzyme loading, in the kinetic constants of their redox catalytic steps, and in their relative ability to transfer electrons to a redox mediator covalently attached to the self-assembled monolayer. The authors concluded that the position of the mutated alpha-helix determined the orientation of the protein with respect to the electrode, and therefore, its ability to establish direct electrical communication. Kanno *et al.*, used a similar immobilization technique using chemical adsorption of Cys residues onto a gold surface [30]. A genetically engineered protein, B5C1, which was tagged with five repeated B domains, each containing a terminal Cys residue, was prepared. The native B5 protein lacked these residues. The modified protein was immobilized to the Au plate via thiol linkages. An immunoassay was performed using the immobilized B5C1 and its corresponding IgG antibody. It was noted that antigen binding activity was considerably higher than that of the adsorbed native species, which lacked the Cys residues. Although this experiment did not utilize an enzymatic biosensor, it does demonstrate that favorable conformations can be achieved using genetic tagging.

Both of the previously mentioned studies involved enzyme immobilization onto a bulk gold surface substrate. Although neither study includes immobilization onto GNPs, both schemes demonstrate a potentially effective method for oriented immobilization onto such a nano-substrate. In 2005, Ha *et al.*, demonstrated feasibility for oriented immobilization onto gold nanoparticles [31]. Tagging esterases with a 6-membered His or Arg tail allowed selective immobilization onto GNPs, resulting in increased bioactivity. For reproducible binding of the enzymes with an un-restricted orientation, gold nanoparticles were prepared via surface modification with 16-mercaptohexadecanoic acid. The carboxylated GNPs functioned as a “nano-supporter”, selectively immobilizing the recombinant esterases through electrostatic affinity with the recombinant tails. Although all the esterases (tagged and untagged) tended to non-specifically adsorb onto the GNP-COOH, the magnitude was strongly dependent on the presence of an appropriate affinity tag. The catalysis of the esterases was investigated by monitoring the UV/Vis absorption peaks of the enzymatic substrate, *p*-nitrophenol butyrate, which develops a new band at 400 nm as it dissociates. The results indicated that the tagged enzymes, specifically the Arg-tagged species, retained significantly more bioactivity than the untagged and His-tagged species. Additionally, the enzyme loading was considerably higher. Higher enzyme loading allows wider linear ranges for signal as enzymes are less likely to become saturated with their substrate at high sample concentrations.

#### 3.2.3. Self-Assembling Monolayers (SAMs)

SAMs provide a simple and well-studied method of immobilizing gold nanoparticles and enzymes onto electrodes, allowing a high degree of control of the composition and thickness of the transducer surface [32]. Colloidal gold-modified electrodes can be prepared by covalently tethering the gold nanoparticles with surface functional groups (–CN, –NH_2_, or –SH) of SAMs-modified electrode surface; alkanethiols are the most intensely studied. Short-chain molecules such as 3-mercaptoproprionic acid and cystamine can be self-assembled on the modified electrode for further nanoparticle binding [9]. These molecules also provide the functional groups necessary for covalent immobilization of the enzyme. Zhang *et al.*, utilized SAMs-modified electrodes when constructing his GOx-based biosensor [33]. The authors used a dithiol spacer molecule to bind gold nanoparticles to the gold electrode surface. The modified electrode was then treated with cystamine which functionalized the gold nanoparticles with terminal amino groups. These amino groups reacted with the aldehyde groups of oxidized GOx enzyme, yielding covalent attachment via imine bonds. A similar process of immobilization was demonstrated by Jia *et al.*, with horseradish peroxidase (HRP) [34]. A gold electrode was first immersed in a hydrolyzed (3-mercaptopropyl)-trimethoxysilane sol− gel solution to assemble a three-dimensional silica gel monolayer. Then gold nanoparticles were chemisorbed onto the –SH groups of the sol–gel monolayer. Finally, horseradish HPR was adsorbed onto the surface of the gold nanoparticles.

Further variation can be achieved using a binary SAM layout. Mixed SAMs of long and short length are reported to show better electron transfer rates than singular component SAMs because of the increased flexibility of redox species distribution at the interface [35]. Park *et al.*, investigated the use of a hetero-length binary SAM layout, utilizing gold nanoparticles and HRP [35]. The gold electrode surface was modified with mixed SAMs, onto which colloidal gold nanoparticles were immobilized. HRP was immobilized on the colloidal gold surface to form a binary biosensor matrix. After the deposition of gold nanoparticles on the gold surface, the GNP-deposited gold electrode and a bare electrode were compared for the surface area and electric current using AFM and cyclic voltammetry. The GNPs strongly adhered to the surface of the gold electrode, had uniform distribution, and were quite stable. A mixed SAM layout, composed of two monolayer molecules—dithiobis-N-succinimidyl propionate (DTSP) and tetradecane-1-thiol (TDT)—was formed utilizing reductive desorption, and cyclic voltammetry was used to verify the formation of mixed SAM. 3-Mercaptopropionic acid (MPA) and TDT were deposited with a specific deposition ratio between the two molecules. MPA was desorbed by applying an electric field to the surface. Next, DTSP was deposited where the MPA was. Ratios of 20:80 and 50:50 between MPA and TDT, respectively, were examined, using cyclic voltammetry. The authors concluded that the ratio of SAM molecules affected the electron resistance. Indeed, the 50:50 SAM showed no oxidation or reduction peaks, suggesting the absence of major pinholes and vacancies in the monolayer. Redox could not readily occur at the electrode surface. However, when the ratio was decreased to 20:80, redox reversibility peaks appeared, indicating that TDT has a higher chain-chain interaction, and were more densely immobilized on the gold surface. The authors concluded that the 20:80 ratio offered decreased electron resistance, compared to the 50:50 ratio. This study suggests that mixed monolayers can provide differing functionality. However, further experimentation is needed to ascertain the optimum ratio of the correct SAM molecules.

Moreover, mixed SAMs provide different functional groups which can confer a higher control of enzyme immobilization. Further enzyme immobilization control can be conferred by a judicious choice of SAM molecules. The objective is to covalently bind enzymes so as to not deter their biological activity or stability. Abad *et al.*, present a strategy for the covalent immobilization of glycosylated enzymes via a binary SAMs layout for a gold macroelectrode or colloidal substrate [36]. Boronic acids, which form cyclic esters with sugars, are incorporated into the SAMs to weakly adsorb the glycoprotein onto the electrode surface via interaction with the sugar groups. To prevent protein release from the electrode surface, they combine the affinity motif of boronates with the reactivity of epoxy groups to covalently link the protein to hetero-functional boronateepoxy SAMs. The concept behind this strategy is the increased immobilization rate achieved by the weak interaction-induced proximity effect between the slow reacting oxyrane groups in the SAM and the nucleophilic residues from the enzyme, allowing the formation of very stable covalent bonds. This concept is exemplified by the use of phenylboronates-oxyrane mixed monolayers, immobilized on a gold substrate via a thiol linkage, as a reactive support for horse radish peroxidase (HRP). It was demonstrated that HRP, with its native glycosylated groups, has a significantly higher immobilization rate than a recombinant HRP which lacked its sugar groups. Therefore, the authors concluded the additional affinity, imparted by the boronate-sugar interaction, greatly increased the immobilization rate.

Multi-layer motifs afford additional control. They are basically an assembly of multiple SAMS, “stacked” on top of each other. This layer-by-layer layout is attractive because of its simplicity of procedure, wide selection of composition, and thickness of the self-assembled layer [37]. Such a layout was prepared by Yang *et al.*[38]. Construction of the multilayer film consisted of glucose oxidase and gold nanoparticles, using cysteamine as a cross-linker based on two covalent reactions: Schiff bases reaction between aldehyde-group of IO_4_-oxidized GOD and amino-group of cysteamine, and the covalent bond between gold nanoparticles (GNPs) and –SH of cysteamine.

#### 3.2.4. Co-Modification with Electrode Matrix

This strategy involves the co-immobilization of the gold nanoparticles and the enzyme into a composite material which will transfer the electric signal from the active site to the electrode, via the polymer-bound colloidal gold. Although SAMs offer many advantages, they are restricted for two principle reasons. They typically form very compact layers which restrict the diffusion rate of reagents due to overcrowding of enzymes and causes steric hindrance about the active site which limits the bioactivity [39]. Unfortunately, the density of GNPs is difficult to control when using a SAM layout. However, using composites allows greater and easier control of the relative amounts and dispersion of the nano-scale species, leading to a lower enzyme density [39].

A nanostructured composite or nanocomposite results when the length of scale of at least one of the components is in the nanometer range [39]. As shown before, nanoparticles of gold possess many favorable qualities such as high surface-volume ratio and electric conductivity. Moreover, due to the nanoparticles dispersion in the composite matrix, it is easier to modify certain properties of the transducer while keeping others. In short, nanocomposites of GNPs and the polymer phase can be made to retain qualities of both. Whereas SAMs are problematic in that they produce a layer of highly packed GNPs leading to overcrowding, such dispersion can be easily controlled using composites, as demonstrated in a percolation curve.

A conductive composite is manufactured if at least one of the phases is an electric conductor. The electrical properties of the composite are determined by the nature, distribution, and relative quantities of the conducting phase, such as the GNPs [39].

A polymer composite, as the name implies, results if at least one of the phases is a polymer—either conducting or non-conducting. In either case, conduction can be imparted or enhanced by incorporating GNPs or other conductive fillers into the matrix.

Conducting polymers are basically organic conjugated polymers, offering many useful electrochemical characteristics such as low ionization potentials, high electric conductivity, and high electronic affinity. This is due to the conjugate pi structure. They can act as metal conductors or as semi-conducting inorganic substrates [39]. Two common conducting polymers include polypyrrole and graphite [40–42].

Non-conducting polymers are polymeric binders, such as epoxy, methacrylate, silicone, or araldite, which are used to impart a certain physical, chemical, or biological stability to the matrix [39]. In short, they “glue” the conducting particles together.

Carbon-based polymers such as graphite have excellent electric properties because of their sp^2^ hybridization; the pi bonds allow rapid electron transfer. Graphite is an ideal conductor phase due to its chemical inertness, wide range of working potentials, low electric resistance (10^−4^ ohms·cm), and low residual currents [39]. One of the simplest composites is based on soft carbon paste. These pastes are produced by mixing a nonreactive conductor such as graphite powder with a nonconductive liquid such as paraffin oil, silicone, or mineral oil (Nujol). Unfortunately, these composites have limited mechanical and physical stability. They degenerate rapidly in flow systems and may be dissolved in non-electrolytic, non-polar solvents [39]. However, overall, co-modification provides an easy and simple way to manufacture a reagent-less biosensor, combining the advantages of colloidal gold and composite materials.

Liu *et al.*, demonstrated a simple and elegant method to create a reagent-less glucose biosensor, based on a colloidal gold-modified carbon-based electrode [40]. Briefly, graphite powder was introduced into a colloidal gold solution and mixed thoroughly. After incubation, the mixture was added to paraffin oil, creating the modified composite electrode. Electrical contact was established by inserting a copper wire into the matrix. The GOx enzyme was immobilized on the electrode by adsorption onto the surface. A limitation of carbon paste is that oxygen is fairly insoluble in it. For an enzyme such as GOx, this is quite disadvantageous. However, this was addressed by the GOx being adsorbed onto the surface of the electrode. Alternatively, the enzyme can be mixed in with the electrode matrix. In a separate experiment, Liu *et al.* manufactured a phenol-detecting biosensor using tyrosinase [41]. The procedure was virtually identical to the previous experiment, except the enzyme was mixed in the carbon paste before curing, yielding a heterogeneous suspension of enzyme. Although the oxidation of phenol requires oxygen, the biosensor retained its bioactivity and provided adequate performance. It was also demonstrated that the incorporation of colloidal gold enhanced the detection limit by 4.25 times, when compared to a reagent-less carbon paste electrode.

Additionally, carbon-based composites can be modified or replaced with other conductive polymers. As stated before, conductive polymers require pi conjugation and such an example is polypyrrole. Indeed, Miao *et al.*, manufactured a GNP/polypyrrole (PPy) biosensor, measuring the electro-catalytic reduction of O_2_ by laccase [42]. Colloids of gold/polypyrrole (AuPPy) composite nanoparticles were prepared by oxidizing PPy with HAuCl_4_ in cetyltrimethylammonium bromide (CBAT) solution. 0.01 μL of pyrrole was transferred to 2 mL 0.5 M H_2_SO_4_ solution with 10 mM CTAB. Then, 10 μL 2 mM HAuCl_4_ solution was added. The gold disk electrodes were mechanically polished with alumina slurries. 20 μL of GNP/PPy colloid solution was cast on the surface of the gold electrodes. For preparation of the laccase electrode, 2 μL 10 mg/mL laccase solution was added to the GNP/PPy-modified electrode and dried. Then 1 μL 0.1% glutaraldehyde was added to the electrode surface and allowed to dry at room temperature. For comparison, a control electrode of laccase on a bare gold electrode was prepared. A 2 μL 10 mg/mL laccase solution was added onto the bare Au electrode and dried, then 1 μL 0.1% glutaraldehyde was added and allowed to dry at room temperature. Cyclic voltammetry revealed a pair of redox peaks at 0.27 V and 0.40 V, respectively. In contrast, the bare gold-laccase control electrode gave no obvious electrochemical signal, indicating that the laccase was not immobilized onto the bare gold surface. Therefore, the redox peaks resulted from the redox reactions of laccase, immobilized on GNP/PPy modified electrode. The authors concluded that the GNP/PPy nanoparticles played an important role in improving the laccase immobilization and facilitating the direct electron transfer between laccase and Au electrode. Table 1 summarizes the analytical figures of merit for several gold nanoparticle containing biosensors with electrochemical detection.

## 4. Carbon Nanotubes (CNTs)

A tremendous amount of research has been performed on the physical and chemical properties of carbon nanomaterials since the discovery of carbon nanotubes by Iijima in 1991 [43]. Iijima produced the first CNTs using an arc-discharge evaporation method.The development of biosensor devices containing these nanomaterials has emerged as the most promising short-term application of CNTs and is an active area of research in physical sciences, engineering, and medicine. The first CNT-based sensor was reported by Britto *et al.*, in 1996 [44]. Since then, CNTs have been incorporated into various electrochemical biosensors because these sensors tend to have higher sensitivities, faster response times and lower detection limits compared to conventional sensor designs with carbon electrodes [9,45]. Due to their high conductivity, fast electron transfer rates and other desirable chemical and physical properties, CNTs have often been used as intermediates between glassy carbon, gold or platinum electrodes and enzyme biorecognition components. For example, in glucose sensors the improved electrocatalytic properties of these nanomaterials effectively lowers the oxidation overpotential for the indirect detection of glucose by H_2_O_2_ oxidation and creates conditions that are favorable for the discrimination of H_2_O_2_ from common interfering species such as ascorbic acid. Biofunctionalization of CNTs confers additional selectivity of detection on the CNTs [45]. The three dimensional shape and large surface area of CNTs allow large enzyme loading that is accessible within a very thin layer [9]. CNTs are also popular in sensor applications other than electrochemical biosensors due to their unique optical, chemical, thermal and mechanical properties [9].

### 4.1. Characteristics of CNTs

CNTs are fullerene-related molecules, composed of graphene sheets which are wound into a cylindrical shape. They may be closed at either end with caps containing pentagonal rings, or they may be left open. Multi-wall carbon nanotubes (MWCNTs) follow the same layout as single-walled CNTs (SWCNTs), except there are multiple layers of CNTs, each enclosing each other [46]. As stated previously, graphite is sp^2^ hybridized, imparting an amazing tensile strength around 50 times more than steel [47]. CNTs are very stiff and have a high strain to failure. Each carbon is covalently bound to its three adjacent neighbors resulting in a seamless structure with hexagonal honeycomb lattices [9]. The hybridization imparts many unique electrochemical characteristics, capable of acting as metallic or semi-conducting depending on their structure. SWCNTs are approximately 1 to 2 nm in diameter, while MWCNTs can range from 2 to 50 nm with an interlayer distance of 0.34 nm. CNTs can be up to hundreds of microns long. MWCNTs are made of several layers of graphitic cylinders, which are centrally nested like the rings of a tree trunk. They are regarded entirely as metallic conductors, which in some regards, makes them better for electrochemical biosensors. However, SWCNTs are more well-defined layouts, allowing their electrochemical properties to be easily understood. SWNTs are more challenging to manipulate for sensor device fabrication than some other nanomaterials. Other limitations of SWCNTs include being too small to interface with large biorecognition components such as cells or tissues as well as not being easy to bio-functionalize. Although the electrochemical properties of both types of carbon nanotubes are not yet fully understood, these materials serve as good candidates for inclusion in amperometric biosensor devices. Electrodes incorporating single or multi-walled CNTs have been found to have fast electron transfer rates as compared to that found for traditional catalytic electrochemical biosensors [9]. Electronic changes in the behavior of SWCNTs have been reported when they interact with proteins and other biologically relevant molecules [48–53].

### 4.2. Preparation Methods for CNTs

**Figure 6.**
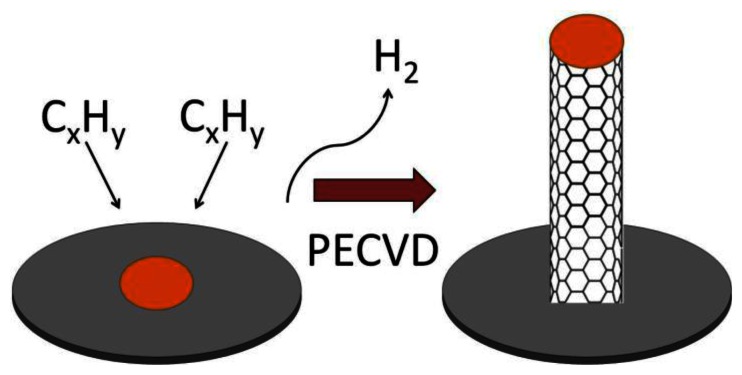
Direct and controlled CNT growth on a catalyst coated substrate using plasma-enhanced chemical vapor deposition (PECVD) method.

The three most common methods for producing CNTs are electric arc discharge (EAD) [54], laser vaporization of a graphite electrode [55] or laser ablation (LA) [56], and chemical vapor deposition (CVD) [57–59]. The method can be chosen carefully to produce CNTs with different properties and forms. EAD uses a direct current arc between two carbon electrodes under an inert atmosphere such as helium or argon gas [43,54]. The electrodes are doped with a suitable catalyst to grow SWCNTs. The CNTs produced by this method are of high quality but vary in diameter and length and may be tangled. In LA, graphite is vaporized by laser irradiation under flowing inert atmosphere at temperatures near 1,200 °C [55,60]. Gas phase hydrocarbon species accumulate on a water-cooled, metal containing collector. Materials, produced using the LA method, are in the form of porous membranes or powders which both require further processing. CNTs produced by LA were more uniform and had a greater tendency to form aligned bundles. In CVD, CNTs are manufactured from the catalytic deposition of hydrocarbon gas, which dissociates either thermally (thermal CVD) or in high energy plasma (plasma-enhanced chemical vapor deposition, PECVD). In thermal CVD hydrocarbon gas at around 700 to 1,000 °C flows over a specific metal substrate such as iron, cobalt, or nickel at high temperatures, with sequential release of H_2_ leaving a graphite network of carbon atoms. The disadvantages of thermal CVD include not being able to use some substrate materials such as glass due to the high temperatures that are required for the method. Also, the CNTs that are produced tend to be randomly oriented and not straight [57]. In PECVD, hydrocarbon gas is introduced into a reactor chamber containing the metal coated substrate surface for CNT growth after atmospheric gases have been evacuated and the substrate is heated to 450–700 °C (Figure 6). A high voltage is applied to the electrode causing ionization of the gases and the formation of plasma. The plasma can also be created using microwaves, radio frequency, inductively coupled PECVD, and dc glow discharge PECVD. The CNT growth rate and diameter can be controlled in PECVD. Of the three techniques, CVD is the most promising because the catalysis-involved process requires a lower temperature than the other two processes and the CNTs can be directly “grown” onto a substrate. CVD allows the location of CNTs to be precisely controlled. CNT arrays can be grown on different substrates and in different patterns allowing the fabrication of a variety of electrochemical biosensors. Also, the resulting CNTs produced by PECVD are straight and aligned vertically in the direction of the electric field [57].

### 4.3. Advantages of CNTs

As a nanoparticle, CNTs have many of the same advantages as GNPs. For instance, they have a high surface-volume ratio, a high electro-catalytic effect, and a fast electron-transfer rate. Although, CNTs are not metal, the hybridization imparts on them enhanced conductive and mechanical properties. Additional advantages include those listed for graphite under nanocomposites for GNPs. CNTs are chemically inert and thermally stable up to 2,800 °C under vacuum and are twice as thermally conductive as diamond [61]. The current carrying capacity is an astounding 1,000 times greater than that of copper wire [62]. Unlike GNPs, CNTs can be assembled into a collection of parallel nanoelectrodes, effectively summing up the individual electric signals into an enhanced, detectable signal [63]. However, to fully manipulate this unique property, low-density, aligned CNTs have to be assembled. The increased spacing prevents diffusion layer overlap with the neighboring electrode, allowing each CNT to act as an individual nanoelectrode, each of which contributes to the observed signal. Also, nanoelectrodes, as opposed to macroelectrodes, allow radial diffusion to occur which increases the flow of reagents to the immobilized enzymes, increases the S/N ratio and lowers the detection limit. Moreover, because of their hollow structure, enzyme loading can be substantially increased through immobilization on the outside and inside of the CNT. This results in much wider linear ranges due to the enzyme active sites not becoming the limiting reagent in the biocatalytic reaction. Several authors have reported that the small size of carbon nanotubes when used in glucose biosensors offers the potential that these materials can penetrate the Glucose oxidase structure without disrupting its catalytic activity thereby allowing for the direct electron transport to the FAD active site of the enzyme [8]. As described previously, the distance between the redox site on the enzyme and the electrode surface as well as the orientation of the immobilized enzyme are critical for efficient electron transfer. CNTs and other nanostructures are able to act as electronic wires that shorten the electron transfer distance and enhance the electron transfer efficiency. Recent studies have also demonstrated that CNTs can enhance the electrochemical reactivity of proteins or enzymes while retaining their biocatalytic activity [45,64].

Given the many advantages offered by CNTs, it is beneficial to explore how they are used in biosensors; specifically, the methods by which the bio-recognition layer (enzyme) is immobilized onto these nano-structures. It is vital that the proteins can be immobilized onto the CNTs while retaining their native biological structure and function. However, it is equally important to study the techniques by which CNTs are immobilized onto the electrode: dispersion and stabilization by oxidative acids, utilization of solubilization media, adsorption, dispersion by surfactant interaction, functionalization, and incorporation into a composite.

### 4.4. CNT-Based Biosensor Fabrication

#### 4.4.1. Dispersion and Stabilization by Oxidative Acids

Although well-ordered, all-carbon hollow CNTs are excellent candidates for biosensors, but they have two major limitations imparted by their hydrophobic nature. These include spontaneous coagulation and lack of solubility in aqueous media [65]. To address this, prepared CNTs undergo oxidative acid treatment which includes refluxing and sonication in a concentrated mixture of sulfuric and nitric acid. Although this procedure can produce defects on the surface of CNTs and shorten the nanotubes, it produces carboxylated sites on the CNT walls and caps, allowing the CNTs to form a dispersed suspension in aqueous media [9]. Using the –COOH groups, the CNTs can be chemically adsorbed onto an electrode surface. A dark stable suspension can be achieved after immobilization via removal of the excess carboxylic acid groups. Kovtyukhova *et al.*, developed a novel method for immobilization of SWCNTs using an oxidative technique previously developed for transformation of graphite to graphite oxide [66]. This process involved treatment with a H_2_SO_4_ containing (NH_4_)_2_S_2_O_8_ and P_2_O_5_ solution, followed by H_2_SO_4_ and KMnO_4_. Oxidation resulted in exfoliation of CNT ropes, ranging from 40 to 500 nm long. The oxidized CNTs slowly formed hydrogels at low concentration (≥0.3 wt%). The authors attributed this to the formation of a hydrogen-bonded nanotube network. The oxidized tubes bonded readily to amine-coated surfaces, on which they adsorbed as a single-layer film.

#### 4.4.2. CNT Adsorption on the Transducer Substrate

To prevent the coagulation that occurs when CNTs are placed in aqueous media, dissolving them in non-polar organic solvents such as N,N-dimethylformamide (DMF) or chloroform followed by sonication allows the formation of homogeneous CNT dispersions that can be used to drop-cast or spin coat transducer surfaces [67]. The solvent quickly evaporates leaving behind a porous, 3-D structure of CNTs on the electrode surface to which the biomolecules can be immobilized. These methods are very popular for CNT immobilization due to their ease and simplicity. The major limitation of adsorption immobilization is the resulting random distribution of nanomaterials that is not reproducible on the transducer surface. The most common subtrates are gold, platinum, glassy carbon, carbon fiber, and glass [9].

Baj-Rossi *et al.*, prepared a biosensor for electrochemical detection of anti-cancer drugs in human serum using chloroform solubilization followed by sonication and drop-casting of MWCNTs with diameter of 10 nm, length of 1–2 μm, and 5%–COOH groups content [67]. The CNTs were directly immobilized onto screen printed graphite working electrodes. Three different cytochrome P450 isoforms were allowed to adsorb onto MWCNTs. Cyclic voltammetry (CV) was performed in phosphate buffer saline (PBS) as well as in human serum to which therapeutic levels of anti-cancer drugs were gradually added. CV gave well-defined current responses upon addition of increasing concentrations of the following anti-cancer drugs: cyclophosphamide, ifosfamide, ftorafur and etoposide. The results show sensitivities in the range of 8–925 nA/μM and detection limits in the range of 0.05–4.9 μM in PBS buffer and 0.5–40 μM in serum [67]. The authors demonstrated that simultaneous detection of two drugs can be achieved with a careful selection of the isoform as enzyme probe according to the drug to be detected.

#### 4.4.3. Dispersion by Surfactant Interaction

Multiple groups have explored noncovalent immobilization methods which preserve the intact CNT structures after their dispersion. The nanostructures were first centrifuged, filtered, distilled, and sonicated followed by a simple noncovalent immobilization by spin coating, evaporation or casting onto the sensor surface [44,68]. However, dispersing and anchoring the CNTs onto the sensor surface in a controlled manner can be challenging due to the hydrophobic properties of the nanostructures [9]. Noncovalent surfactant- and polymer assisted aqueous dispersion which utilize the hydrophilic caps of CNTs have helped overcome some of the limitations seen with simple physical stabilization [69–71].

#### 4.4.4. Surface Functionalization

This method requires covalent modification of the CNT and/or electrode surface with functional groups that will bind) to the electrode or substrate surface. The modification of CNTs usually involves the ends, sidewalls, or defects which result from the oxidative acid pretreatment of CNTs and are rich in CNT-bound carboxylic groups [9]. The linkages between the functional components and CNTs, which may or may not involve coupling agents, are typically based on carboxylate chemistry via amidation and esterification therefore involving covalent bonding or alternatively ionic interactions that are noncovalent in nature.Liu *et al.*, provided a method by which SWCNTs were covalently self-assembled onto a gold electrode surface [72]. The authors used dicyclohexylcarbodiimide (DDC), a coupling agent that transforms the carboxylated ends of the CNTs to carbodiimide leaving groups, to react with cysteamine (NH_2_CH_2_CH_2_SH). The resulting CNTs had a free thiol group which readily reacted with the gold substrate, forming a covalent linkage. Atomic force microscopy (AFM) images revealed that the nanotubes had been immobilized on gold substrate, forming a self-assembled monolayer structure with a perpendicular orientation. This method offers control of the spatial distribution, length, and surface patterns, by adjusting the assembled amount and time.

Moreover, activating CNT surfaces is important in order to improve the performance of the prepared biosensors. The external added molecules can be as small as simple amino acids or as large as protein macromolecules. CNT solubilization in aqueous media is important for use of CNTs as supporting matrix for the immobilization of proteins. This can be achieved by the surface functionalization of CNTs with ionic, hydrophilic groups, or with water-soluble polymers. Soluble CNTs have been shown to have electronic properties similar to CNTs that were not functionalized [73]. The electronic properties of the CNTs seem to depend primarily on the nanotube's diameter and chirality. In 2008, Yan *et al.*, demonstrated a method, whereby the CNT was modified by covalent bonding of polyethylene imine or poly(acrylic acid) (PAA) to obtain water-soluble MWCNTs [74]. In 2009, Cui *et al.*, produced MWCNTs, modified by redox polymer, poly(vinylimidazole) complexed with Os(4,4′dimethylbpy)2Cl(PVI-demeOs), resulting in the transformation of the MWCNT surface from hydrophobic to hydrophilic [75]. The biosensor showed enhanced sensing sensitivities induced by the redox polymer film.

Park *et al.*, immobilized D-(+)-galactose on SWCNTs functionalized with –COCl without causing any major structural alterations in the nanomaterials [76]. The D-(+)-galactose conjugated SWCNTs were then dropped onto the surface of a SiO_2_ substrate to fabricate molybdenum (Mo) electrodes that were used in the prepared electrochemical biosensor for the detection of galectin-3, a cancer marker. The electrochemical response of the D-(+)-galactose-conjugated SWCNTs differed significantly between the samples with and without galectin-3 [76]. Therefore, these modified CNTs can potentially be useful in electrochemical biosensors for the detection of galectin-3.

#### 4.4.5. Incorporation into a Composite

Perhaps, the easiest and most popular method of CNT immobilization is the incorporation of the nanomaterial into a composite. The first nanotube composites were manufactured by Ajayan *et al.*, in 1994 by mechanically mixing MWCNTs and an epoxy resin [77]. A composite mixture of CNTs and pi-conjugated polymers such as graphite can be viewed as an extreme form of a conducting polymer, offering a high surface area-volume ratio and enhanced electronic properties. Wallace *et al.*, produced a GOx-based biosensor by embedding MWCNTs into polypyrrole phase with 0.1 M NaClO_4_ as a supporting electrolyte [78]. The biosensor retained 70% of its stability after 3 days storage in dry phase at 4 degrees Celsius. Wang *et al.*, were the first to use CNTs in fabrication of needle-like microsensors for glucose in 2003 [79]. A mixture of GOx and CNT was packed within small polyimide tubing and coated with a Nafion film at the end of the sensor.

Jia *et al.*, prepared and optimized a needle type biosensors for glucose using composite of MWCNTs, graphite powder, and freeze-dried powder of GOx inside a glass capillary [80]. MWCNTs with average length of 20 μm and a mean diameter of 15 nm were treated with strong acid and agitated. MWCNTs were then filtered, rinsed with water, and dried in an oven. The acid treated MWCNTs, GOx and graphite powder were mixed into a paste and pressed into the cavity at the end of a glass capillary containing a copper wire. Finally, the end surface of the electrode was soaked in paraffin, oven dried, and polished to a smooth surface with weighing paper. The composition ratio of MWCNTs mixture to GOx was found to be critical for current response. The biosensor had good sensitivity and stability, and a detected range of up to 20 mM glucose. The current response of the biosensor decreased by less than 10% during 24 hours on continuous online monitoring of glucose and was down to 65% after two weeks [80].

#### 4.4.6. Carbon Nanotube Array Biosensors

CNT arrays consist of vertically aligned bundles of relatively short CNTs. CNT arrays have many of the same desirable properties that were observed for individual CNTs such as good electrical conductivity and efficient electron transfer reactions [6]. Direct electron transfer between redox active enzymes such as Glucose oxidase and CNT arrays has been reported [81]. However, they may not be robust requiring the use of a polymer or a glass casing as a protective outside support. UV curing polymers and epoxy resin followed by m-phenylenediamine hardener have been used as an outside coating or a layer for depositing CNT arrays [82,83]. CNT arrays can be prepared from SWCNTs using oxidative acid treatment as described in Section 4.2. CNT arrays can also be grown directly onto an electrode surface in a controlled manner by using CVD or plasma-enhanced CVD methods [57,84].

Carbon nanotube needle biosensors can be prepared in a cost effective manner by welding a bundle of MWCNTs in an inert atmosphere onto the tip of a tungsten needle under a bright field microscope [82]. The needle can later be encased in glass and a UV curing polymer coating to electrically insulate the tugsten needle leaving only the tip exposed to the analyte [82]. The bundle of nanotubes at the tip of the transducer may be sharpened using acid etching or electrical discharge to further lower the sensor detection limits. Yun *et al.*, demonstrated a relatively simple manufacturing process can be used to prepare enzyme-based nanosensors for analytes such as glucose.

### 4.5. Immobilization of Enzyme onto CNT

Immobilization of enzyme onto a CNT-modified electrode is of great importance, given the aforementioned advantages of incorporating them into a biosensor. In this review, two main enzyme immobilization methods onto CNT-based sensors, physical adsorption and chemical cross-linking, will be discussed.

#### 4.5.1. Physical Adsorption

Physical adsorption utilizes non-covalent methods to attach enzymes to the modified transducer. With GNPs, this was accomplished with electrostatic interactions between the reducing agent such as citrate to the positively charged amino acid residues of the enzyme. However, with CNTs, the aromatic structure is quite hydrophobic and does not lend itself to electrostatic bonding (unless functionalized). Instead, hydrophobic interactions between CNTs and aromatic residues are responsible for physical adsorption. Azamian *et al.*, demonstrated that protein adsorption to CNTs is independent of pI values, suggesting the electronic interactions play a very minor role in physical adsorption of proteins onto CNTs [85]. Lyons and Keeley recently manufactured a GOx-based biosensor utilizing physical adsorption of the enzyme onto a CNT-modified electrode [86]. Electrodes (either gold or glassy carbon) were prepared by mechanical polishing using alumina and nylon pads. A suspension of SWCNTs was prepared by adding SWCNTs to dimethylformamide or *N*-methyl-2-pyrrolidone, followed by sonication. The resulting suspension was cast onto the macroelectrode surface and the organic solvent was evaporated. Solutions of GOx were prepared by adding GOx to a phosphate buffer solution. The enzyme was physically adsorbed on electrode surfaces by drop coating the enzyme solutions and allowing the solvent to evaporate at room temperature. Finally, Nafion was cast and allowed to dry at room temperature. The resulting film covered the entire electrode. AFM images revealed a random orientation of the GOx on the modified electrode. The electrochemical responses of the SWCNT-modified electrodes and bare electrode were determined using cyclic voltammetry. While the bare GC electrode demonstrated a virtually flat voltammetric response, a pair of well-defined redox peaks was observed at the both the SWCNT/GOX and SWCNT/GOx/Nafion-modified electrode. The Nafion was determined to exhibit the best electrochemical response. The authors concluded that this resulted because of the increased solubility of CNTs in a Nafion media.

#### 4.5.2. Chemical Cross-Linking

**Figure 7.**
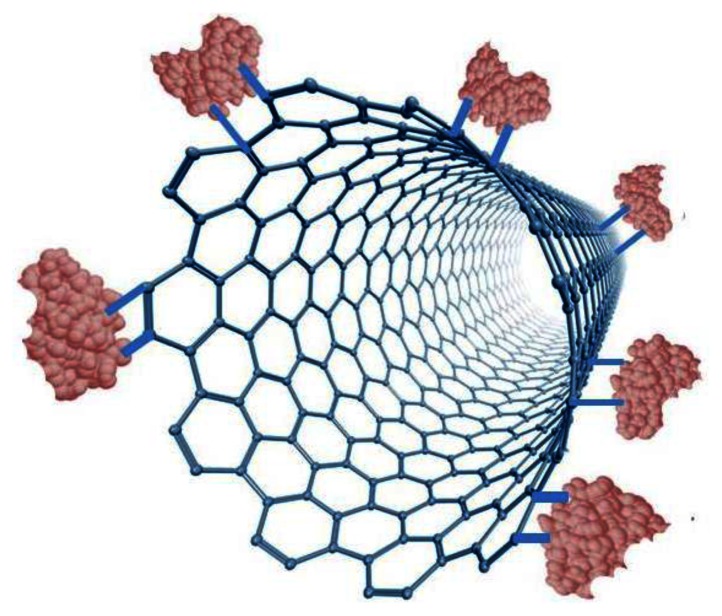
SWCNT with covalently bound enzyme molecules.

Chemical cross-linking involves covalent attachment of the enzyme onto CNT via a linker molecule such as glutaraldehyde (Figure 7). Covalent attachment provides a much stronger attachment method than physical adsorption and may provide the enzyme with a higher catalytic activity. Carpani *et al.*, demonstrated an amperometric GOx biosensor based on cross-linkage to a SWCNT-modified electrode [87]. The glass carbon (GC) electrodes were polished with emery paper and aqueous alumina slurry. Then, the electrode was electrochemically activated to generate an oxide layer. To do this, an oxidative treatment was carried out in a LiClO_4_ solution, and by applying a positive potential. Purified SWCNTs were dispersed in dimethylformamide (DMF) with ultrasonication, resulting in a black CNT suspension. The resulting mixture was dropped onto the GC. Glucose biosensors were prepared by coating the surface of the two kinds of electrodes with enzymatic solution, containing GOx in 0.1 M PBS. The electrodes were allowed to dry in air and were kept in a chamber saturated with the vapors of glutaraldehyde solution at room temperature. The treatment with a cross-linking agent aimed to avoid the enzyme release. Cyclic voltammetry revealed that the CNT-modified electrode gave a similar electric response as the activated glass electrode. Although the activated GC electrode gave a lower detection limit and a higher S/N ratio, the CNT-modified biosensor had a higher sensitivity, attributed to the direct electron transfer between the active site and CNT. The authors concluded that both biosensors gave comparable results.

## 5. Graphene

Graphene, one of the newest nanomaterials used in biosensors, is a two-dimensional one-atom thick sheet made of pure carbon with atoms arranged in a repeating hexagonal pattern similar to graphite. As in graphite, the carbon atoms are sp^2^-hybridized in a densely packed honeycomb crystal lattice. The resulting nanomaterial, which looks like flat chicken wire at the atomic level, was discovered in 2004 [88]. Since graphene possesses the same basic structure as graphite and carbon nanotubes, it has many of the same physical properties. It is biocompatible, has fast electron transport, high thermal conductivity, and high mechanical strength [89]. In addition, Graphene is a zero-gap semiconductor material that is transparent, highly elastic, low cost and environmentally friendly [90] making it an attractive alternative for nanomaterial-based biosensors. Graphene has shown to be promising in chemical and biological sensor applications during the past several years including enzymatic biosensors [89–93]. The structural difference between carbon nanosheets and nanotubes can be utilized for design and fabrication of novel biosensors. Graphene is also easier to functionalize for the immobilization of proteins than CNTs. Graphene has been found to have high catalytic activity with hydrogen peroxide and efficient direct electrochemistry with glucose oxidase making an excellent transducer material for glucose biosensors [93].The electron transfer between graphene and redox active species occurs at the edges of the graphene sheet and/or at defects in the basal plane [90]. Therefore, the high surface area of graphene typically provides a large number of electroactive sites. Ultrathin multilayer graphene nanoplatelets have also been used as transducers in glucose biosensors [94,95].

### 5.1. Preparation Methods for Graphene

Graphene sheets are produced via three main approaches; careful mechanical exfoliation of graphite using adhesive tape, chemical methods, and chemical vapor deposition (CVD). Exfoliation of graphite oxide can also be done [90]. Utilizing adhesive tape to mechanically exfoliate graphite remains the preferred approach, since it results in the best quality and least modified graphene [7]. However, this method can be tedious as the number of graphene layers stuck on the tape surface may be unknown. Chemical methods require utilizing a strong acid to initially oxidize the graphene thus creating a large number of oxygen containing functional groups on the graphene surface [96]. The resulting graphene oxide is hydrophilic and can be dissolved into a single graphene sheet in polar solvents. Then the graphene oxide undergoes reduction by heating it in the presence of a reducing atmosphere or it may be chemically reduced by hydrazine back to graphene. Unfortunately, the disadvantage of this method is that some residual graphene oxide and carbon oxygen bonds may remain on the surface of the sheet [97]. Finally, graphene can be produced by electrochemical reduction of graphene oxide. As discussed previously, in CVD, metallic substrates such as nickel or copper are exposed to hydrocarbon vapors and heated to about 1,000 °C [98]. Achieving monolayers of graphene using CVD continues to present a challenge, however copper remains the most promising substrate for producing graphene monolayers [99].The graphene oxide structure may not be completely planar due to damage to the sp^2^ carbon network caused by the above methods. As stated earlier, oxygen-containing groups on graphene present ideal sites for the immobilization of biomolecules such as enzymes.

### 5.2. Immobilization Methods for Enzymes

#### 5.2.1. Covalent Conjugation of Enzyme to Graphene and Its Derivatives

Liu *et al.*, prepared a glucose biosensor by covalent attachment of glucose oxidase (GOx) to graphene oxide sheets [100]. The covalent attachment was created between the carboxyl acid groups on graphene oxide sheets and the amines of the enzyme in the presence of 1-ethyl-3-(3-dimethylaminoprophy) carbondiimide hydrochloride (EDC) and *N*-hydroxysuccinimide (NHS). The electrochemical performance of the biosensor was evaluated at 0.4 V *vs.* Ag/AgCl using amperometry. The biosensor had a linear range up to 28 mM/mm^2^ glucose and a sensitivity of 8.045 mA/cm^2^M^1^ (as determined from the slope of the calibration curve). The prepared enzyme electrode had good storage stability and reproducibility.

#### 5.2.2. Use of Linker Molecules

Huang *et al.*, prepared biosensors for glucose and glutamate by immobilizing glucose oxidase (GOx) and glutamic dehydrogenase (GluD) onto a graphene film using a linker molecule [101].The graphene device was incubated with 5 mM 1-pyrenebutanoic acid succinimidyl ester (a linker molecule) in dimethylformamide (DMF) for two hours followed by washing. The linker-modified graphene was then incubated with GOx or GluD overnight at 4 °C followed by rinsing with water and buffer. Any excess reactive groups remaining on the surface on the sensor device were blocked with ethanolamine. The detection limits of the graphene-based glucose sensor (0.1 mM) and glutamate sensor (5 μM) were comparable with other commonly used electrochemical biosensors [101] but inferior to some state-of-the-art sensors that are nanomaterial based. The authors hypothesize that graphene biosensors in general, are superior to SWNT-network sensors due to the sensitivity of SWNT network sensors being impaired by the presence of metallic tubes, the functionalization of enzymes on flat graphene film being more effective and uniform than on small carbon nanotubes, and the functionalization steps possibly altering tube-to-tube contacts in the SWNT network sensors [101].

#### 5.2.3. Incorporation of Enzymes into Composite Films

Lu *et al.*, prepared a hydrogen peroxide (H_2_O_2_) biosensor capable of direct electrochemistry between horseradish peroxidase (HRP) and the electrode by utilizing a single-layer graphene nanoplatelet-enzyme composite film [95]. A mixture of HRP, single-layer graphene nanoplatelets (SLGnP), and tetrasodium 1,3,6,8-pyrenetetrasulfonic acid (TPA) was applied to glassy carbon (GC) electrode surface and dried overnight. A drop of Nafion was used to bind the composite film to the electrode surface. The graphene and enzyme interaction was studied using scanning electron microscopy (SEM). Ultraviolet visible spectroscopy was also performed to confirm that the immobilized HRP retained its secondary structure after incorporation in the composite film. The electrocatalytic reduction of H_2_O_2_ at the composite film modified GC electrode was quite rapid and efficient as indicated by the amperometric responses in nA scale which reached steady state current in less than 1 second [95]. This mediator-free design, which may be adapted for other enzymes, seems promising for fabrication of new biosensors.

Shan *et al.*, prepared electrochemical glucose sensors with polyvinylpyrrolidone-protected graphene/polyethylenimine-functionalized ionic liquid (PFIL)/GOx [102]. The carboxyl terminated ionic liquid was covalently attached to polyethyleneimine. The films have been shown to have good stability, wide solubility, high biocompatibility, and high conductivity leading to enhanced electrochemical response. The sensors had linear response range up to 14 mM glucose. Direct electron transfer of GOx was observed and the sensors appeared stable.

Shan *et al.*, also prepared glucose biosensors based on graphene/gold nanoparticles/chitosan nanocomposites film [103]. These sensors had linear response ranging from 2 to 10 mM glucose at − 0.2 V and from 2 to 14 mM at 0.5 V. The biosensors also had good reproducibility and detection limit of 180 μM. The hybrid biosensors containing both gold nanoparticles and graphene also performed well in human blood with linear responses from 2.5 to 7.5 mM [103]. Kang *et al.*, have prepared biosensors with nanocomposite films containing glucose oxidase, graphene, and chitosan for the detection of glucose [104]. The authors observed significantly higher enzyme loading (1.12 × 10^−9^ mol/cm^2^) for the nanocomposite film sensors compared to base glassy carbon electrode surfaces. The linear range was from 0.08 mM to 12 mM glucose with a detection limit of 0.02 mM and high sensitivity of 37.93 μA/mM cm^2^[104]. The biosensors' excellent performance was considered to be the result of large surface-to-volume ratio and high conductivity of graphene. In addition, incorporating chitosan improved the biocompatibility of the sensor and enhanced GOx enzyme absorption.

## 6. Nanowires in Biosensors

Nanowires have also been incorporated into nanoscale electronic biosensor devices in recent years [9]. Most of these nanowires are silicon-based semiconductors, conducting polymers, and oxides. Metallic nanowires have also been used in biosensors. The nanowire material can be tailored for the desired functionality of the sensor device. Nanowires have several attractive features such as; their extremely high sensitivity in detecting surface biointeractions (an advantage of their high aspect ratios) [9]. The electronically switchable properties of semiconducting nanowires allow for direct and label-free electrochemical detection. Though an active area of research, the topic of nanowire-modified biosensors is beyond the scope of this review and will not be discussed in detail.

## 7. Conclusions

Nanomaterials such as gold nanoparticles and carbon nanotubes play an increasingly important role in society, such as being an active, interdisciplinary area of research as well as use in the development of biosensors for important analytes such as glucose, tumor markers, and therapeutic drugs. Incorporation of graphene in electrochemical biosensors has also become more common and appears to hold promise for the construction of inexpensive and stable devices with excellent biocompatability. Hybrid biosensors incorporating more than one type of nanomaterial are also becoming more popular. In addition, the sensitivity and overall performance of biosensors has improved tremendously as a result of incorporating nanomaterials in their construction. Because of their nanoscale dimensions, nanosensor devices are minimally invasive and therefore suitable for many *in-vivo* and *in-situ* measurements. Some nanomaterials also allow direct electron transfer between enzymes and tranducer surface. This review described various nanomaterial containing electrochemical biosensors with emphasis on related immobilization chemistry.

## Figures and Tables

**Table 1.** Summary of gold nanoparticle containing electrochemical biosensors.

**Authors**	**Enzyme Immobilization Method onto GNPs**	**Enzyme**	**Analyte**	**Detection Limit**	**Linear Range**	**Sensitivity**
Z. Liu *et al.*	Chemical Adsorption onto GNPs	Tyrosinase	Catechol	0.06 μM	4.0 × 10^−7^ to 7.0 × 10^−5^ M	3.94 mA·mM^−1^·cm^−2^
S. Zhang *et al.*	Covalent Attachment Utilizing SAMs	Glucose Oxidase	Glucose	8.2 μM	2.0 × 10^−5^ to 5.7 × 10^−3^ M	8.8 μA·mM^−1^·cm^−2^
J. Jia *et al.*	Covalent Attachment onto GNPs to 3D Sol-Gel	HRP	H_2_O_2_	2.0 μM	5.0 × 10^−6^ to 10.0 × 10^−3^ M	
W. Yang *et al.*	Covalent Attachment onto Multilayer Motif	Glucose Oxidase	Glucose		1.0 × 10^−5^ to 1.3 × 10^−2^ M	5.72 mA·mM^−1^·cm^−2^
S. Liu *et al.*	Co-Modification into a Carbon Paste Matrix	Glucose Oxidase	Glucose	0.01 mM	0.04 to 0.28 mM	8.4 mA·mM^−1^·cm^−2^
